# Impact of hepatic vessels on whole liver proton density fat fraction and R2* quantification

**DOI:** 10.1186/s41747-025-00663-1

**Published:** 2026-01-05

**Authors:** Ana Jimenez-Pastor, David Marti-Aguado, Bernardo Pereira, Clara Alfaro-Cervello, Alexandre Perez-Girbes, Angel Alberich-Bayarri, Luis Marti-Bonmati

**Affiliations:** 1 Quantitative Imaging Biomarkers in Medicine, Quibim SL, Valencia, Spain; 2 Digestive Disease Department, Clinic University Hospital of Valencia, INCLIVA Biomedical Research Institute, Valencia, Spain; 3 Faculty of Medicine and Dentistry, University of Valencia, Valencia, Spain; 4 Pathology Department, Clinic University Hospital, INCLIVA Health Research Institute, Valencia, Spain; 5 Biomedical Imaging Research Group (GIBI230), La Fe Health Research Institute and Imaging La Fe node at Distributed Network for Biomedical Imaging (ReDIB) Unique Scientific and Technical Infrastructures (ICTS), Valencia, Spain; 6 Radiology Department, La Fe University and Polytechnic Hospital, Valencia, Spain

**Keywords:** Artificial intelligence, Blood vessels, Fatty liver, Iron overload, Magnetic resonance imaging

## Abstract

**Objectives:**

This study investigated the influence of hepatic vessels on the quantification of magnetic resonance imaging (MRI) proton density fat fraction (PDFF) and R2* using automated whole-liver segmentation.

**Materials and methods:**

This prospective multicenter study included patients with chronic liver disease having paired liver biopsy and MR exams with a standardized multiecho chemical-shift gradient echo sequence. Automated whole-liver segmentation was performed, generating two masks per patient, one including and the other excluding the major hepatic vessels. PDFF and R2* were quantified and graded for both masks. Histological grading of hepatic steatosis and iron overload severity was used as a reference standard.

**Results:**

A total of 377 patients were evaluated, of whom 54% had hepatic steatosis and 20% had iron overload on biopsy readings. Stratified by histological grades, there were no statistically significant differences in the distribution of PDFF or R2* between the two segmentation masks. Overall, PDFF and R2* values were minimally lower when vessels were included, with a bias of -0.06% for PDFF and -0.25 s^-1^ for R2*. A lower coefficient of variation was obtained for both imaging parameters after excluding vessels. Patients were classified in the same PDFF grades despite the segmentation approach, and only 7 cases (1.9% of the study population) were reclassified for R2* grading, all being upgraded after vessel exclusion.

**Conclusion:**

Excluding hepatic vessels entails nonsignificant differences in PDFF and R2* quantification. Although with limited impact, vessel exclusion improves biomarker precision in research settings demanding high accuracy and increases clinicians’ confidence when using automatic tools in clinical practice.

**Relevance statement:**

Fat and iron quantification on MRI are key imaging biomarkers for the accurate non-invasive assessment of patients with chronic liver disease. Proton density, fat fraction, and R2* quantification show minimal differences if hepatic vessels are included or excluded from the liver segmentation mask.

**Key Points:**

- The effect of hepatic vessels on proton density, fat fraction, and R2* quantification was evaluated.
- No significant differences were found, excluding hepatic vessels, although their inclusion showed a small negative bias.
- Vessel exclusion may improve clinicians’ confidence and precision in high-sensitivity applications.

**Graphical Abstract:**

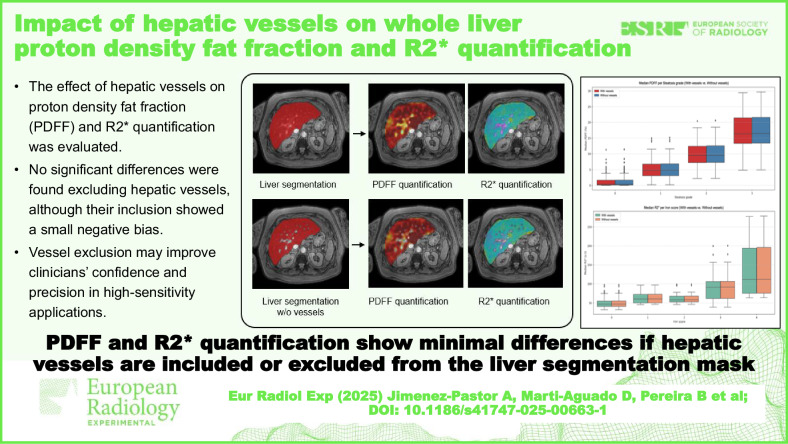

## Introduction

Quantitative liver magnetic resonance imaging (MRI) has become an essential tool in both clinical assessment and research involving patients with chronic liver diseases. The growing burden of conditions such as metabolically-dysfunction-associated steatotic liver disease (MASLD) and alcohol-related liver disease, combined with the limitations of liver biopsy, has fostered the use of noninvasive imaging biomarkers [1, 2]. Multiecho chemical shift-encoded (MECSE) MR acquisitions have enabled accurate and simultaneous quantification of two relevant imaging biomarkers: proton density fat fraction (PDFF), a surrogate for hepatic steatosis, and R2*, a surrogate for iron content [3, 4]. These biomarkers have shown strong associations with histopathological grades, making them attractive tools for diagnosis, disease monitoring, and evaluation of treatment response [4‒6].

Despite their strengths, the reliability of PDFF and R2* quantification mainly depends on the standardization and reproducibility of the liver areas being measured. The first clinical studies relied on regions of interest manually placed in different liver segments to derive PDFF and R2* mean values [7, 8]. Although this approach is relatively straightforward, it has several limitations: (i) it samples only a small portion of the liver; (ii) it is susceptible to operator bias; (iii) it is time-consuming; and (iv) it fails to adequately capture spatial heterogeneity, especially relevant in patients with MASLD where fat and iron distribution may vary significantly across hepatic segments [9, 10]. Furthermore, region-of-interest-based approaches often exclude regions near vascular and biliary structures, underestimating disease burden in those regions.

To overcome these limitations, automated whole-liver segmentation methods have emerged, leveraging image processing techniques and artificial intelligence to delineate the entire liver parenchyma in an efficient and reproducible manner [11]. These methods facilitate comprehensive analysis of the entire parenchyma, and allow large-scale data extraction, which is essential for population studies and longitudinal imaging biomarker analysis. However, the effect of including or excluding the intrahepatic vessels when calculating PDFF and R2* from the entire organ remains unclear.

Hepatic vessels have inherently different image signal characteristics than the parenchyma. Although their inclusion might simplify the clinical workflow by eliminating the need to review vessel exclusion in segmentation masks, it might bias PDFF and R2* measurements, leading to systematic errors. This bias can introduce changes in the quantification of imaging biomarkers, having an impact on clinical management and therapeutic trials [12].

While some segmentation tools attempt to exclude vessels through thresholding or anatomical priors [13, 14], the degree to which their inclusion affects biomarker quantification in real-world patient populations is unknown. While most prior studies have validated PDFF and R2* against histology using whole-liver or region-of-interest-based methods [15, 16], this is the first to assess the impact of intrahepatic large vessels on biomarker precision and variability.

This study aims to address this knowledge gap by evaluating a large, multicenter cohort of patients with chronic liver disease who underwent both liver biopsy and MR imaging. Specifically, we assessed whether the inclusion of hepatic vessels within the segmented liver parenchyma significantly affects PDFF and R2* measurements. We hypothesize that vessel exclusion improves the accuracy of clinical assessment and supports the establishment of standardized imaging protocols and post-processing methodologies.

## Materials and methods

### Study design and population

This prospective multicenter study enrolled adult patients with chronic liver disease who underwent both liver biopsy and MRI within a one-month interval (median time difference of 19, range 13‒26 days). Recruitment occurred between 2017 and 2024 (study registry: 2017/0031/PI) at three medical centers in Valencia, Spain. Inclusion criteria were: ≥ 18-years-old, adequate specimen of liver biopsy for histological evaluation (> 15 mm, > 6 portal tracts), and written informed consent, as approved by the institutional review boards of all participating hospitals. Patients with prior liver transplantation, contraindications to MRI, severe motion or technical artifacts on MRI, decompensated cirrhosis, and the presence of hepatocellular carcinoma, were excluded.

### MRI protocol and image analysis

All participants underwent a standard non-enhanced liver MRI examination with the same scanner (3-T Tx Achieva, Philips Healthcare) at ICTS-ReDIB Imaging La Fe (Valencia, Spain) A two-dimensional transversal MECSE gradient-echo sequence with 12 echoes (echo time 0.9–7.9 ms; delta echo time 0.7 ms; repetition time 9 ms), and low flip angle of 10° to minimize T1 bias, was used for the simultaneous PDFF and R2* quantification analysis. Imaging was performed during breath-hold, with whole-liver coverage; 24‒34 slices were acquired per volume with a slice thickness of 6‒7 mm.

**Fig. 1:**
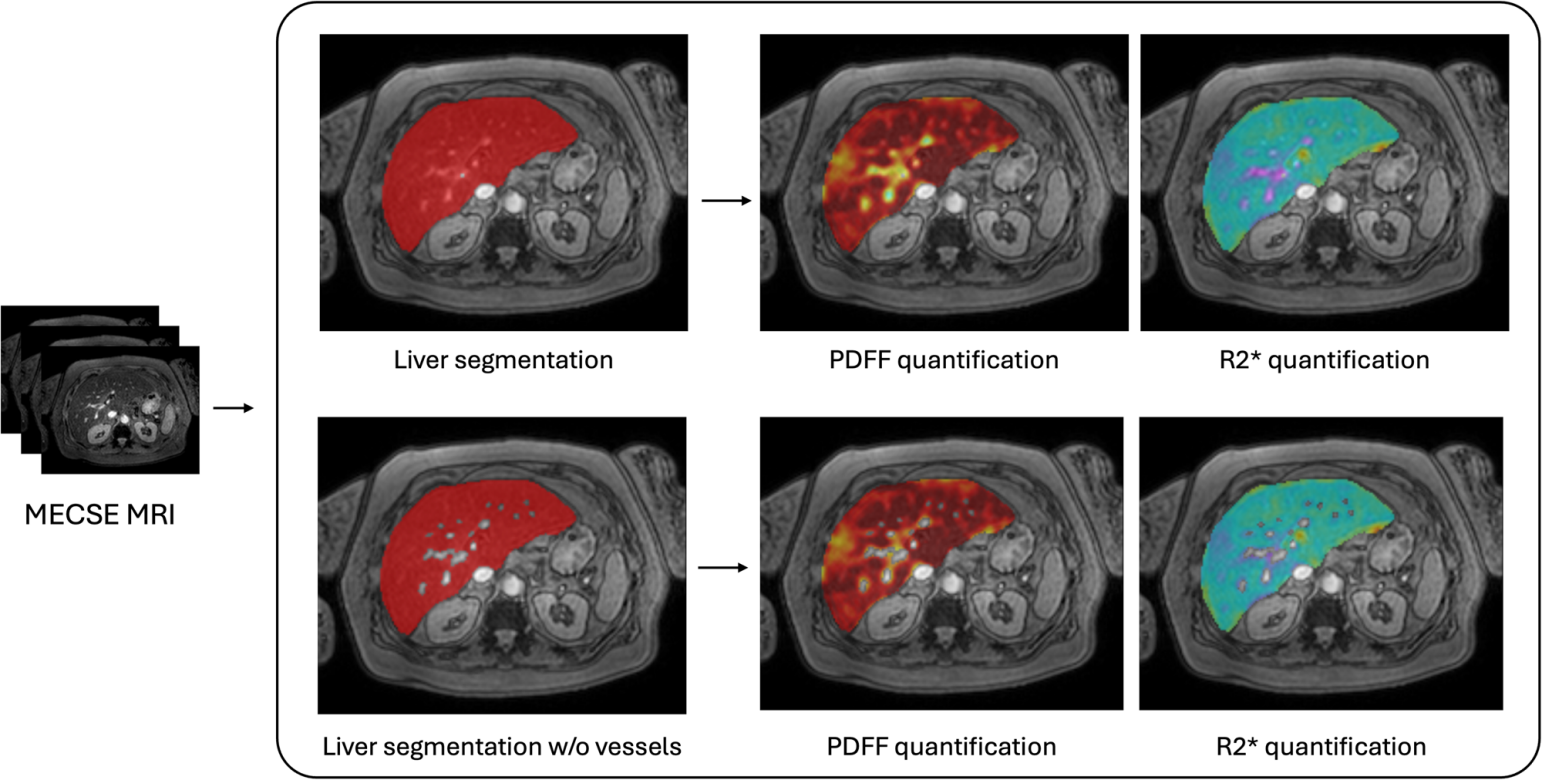
Pipeline followed for image analysis on MECSE images. Whole-liver segmentation, including (top row) and excluding (bottom row) hepatic vessels, was automatically computed. For each, PDFF and R2* parametric maps were calculated. MECSE, Multiecho chemical shift encoded gradient-echo; MRI, Magnetic resonance imaging; PDFF, Proton density fat fraction

Postprocessing was performed using QP-Liver® (Quibim, Valencia, Spain), an artificial intelligence-based software certified (Conformité Européene‒CE mark/UK conformity assessment class IIa) for automated liver segmentation and quantitative biomarker extraction. For each subject, two segmentation strategies were applied (Fig. 1):

1. whole-liver segmentation, including hepatic vessels [11];
2. whole-liver segmentation, excluding hepatic vessels. Vessels were automatically detected through an artificial intelligence-based algorithm and excluded from the liver mask. The vessel's automatic segmentation algorithm was based on a convolutional neural network with the same architecture as already proposed [11]. This algorithm yielded high accuracy when validated against expert manual annotations, with Dice coefficients of 0.97 in the internal test set, 0.93 in an external Siemens 3-T dataset, and 0.85 in an external Philips 1.5-T dataset (details in Supplementary Material 1). This ensures reproducibility and robustness across scanners.

Voxel-wise PDFF (%) and R2* (s⁻¹) maps were computed, and statistical descriptors (median, mean, interquartile range) were extracted for both segmentation masks.

### Histological analysis

Histological evaluation was centrally performed by an experienced liver pathologist (C.A.C, with > 10 years of experience), blinded to clinical data and MRI results. After formalin fixation, samples were embedded in paraffin, and tissue sections were cut and stained with hematoxylin and eosin for steatosis detection and Perls’ stain for iron assessment. Steatosis was graded according to the Nonalcoholic Steatohepatitis‒NASH Clinical Research Network scoring system (S0‒S3) [17]. The grading of iron storage was assessed using the Scheuer scoring system (Fe0‒Fe4) [18].

### Statistical analysis

The primary analysis was planned as a paired two-sided test for equivalence on the mean difference. Because PDFF and R2* were considered co-primary endpoints, a Bonferroni correction was applied (α_total_ = 0.05, α_per-endpoint_ = 0.025). With 90% power (*z*_1−β_ = 1.282) and *z*_1−α_ = 1.96, the required sample size per endpoint was estimated as:

$$n={\left(\frac{{z}_{1-\alpha }+{z}_{1-\beta }}{\Delta }{s}_{d}\right)}^{2}$$

Estimates of the standard deviation of paired differences (*s*_d_) were obtained from reproducibility data in the literature. The Quantitative Imaging Biomarker Alliance (QIBA) PDFF profile reports a repeatability coefficient of 4.7% [19], corresponding to *s*_d_ ≈ 2.4%. For R2*, a *s*_d_ ≈ 5.1 s⁻¹ was assumed based on the repeatability coefficient for confounder-corrected R2* (10 s⁻¹ at 3 T) reported in [4]. With equivalence margins of Δ = 0.5% for PDFF and Δ = 2 s⁻¹ for R2*, the resulting sample size requirements were *n* ≈ 101 for PDFF and *n* ≈ 69 for R2*. Selecting the maximum across endpoints and scenarios yields a conservative requirement of *n* = 101.

Given non-Gaussian distributions, differences between the PDFF and R2* values with the two segmentation methods were assessed using the nonparametric Mann–Whitney *U*-test. The Spearman rank correlation coefficient (ρ) was used to explore the correlation between quantitative metrics computed with both segmentation methods. Statistical significance was set at *p* < 0.05. Median PDFF and R2* values were compared by linear regression and Bland–Altman analyses. Also, the coefficient of variation (CoV) of both PDFF and R2* parametric maps was measured by dividing the standard deviation by the mean value.

Reclassification analysis was conducted by categorizing patients into steatosis and iron overload grades based on PDFF [20] and R2* validated thresholds [15]. We assessed the number of patients who modified their grade severity when using each segmentation approach. The following imaging thresholds were used:

- Steatosis (PDFF): S0 (< 5.75%), S1 (5.75‒15.5%), S2 (15.5–21.35%), and S3 (> 21.35%);
- Iron overload (R2*): Fe0 (< 55 s^-1^), Fe1 (55‒56 s^-1^), Fe2 (56‒64 s^-1^), Fe3 (64‒69 s^-1^), and Fe4 (> 69 s^-1^).

Analyses were performed using Python 3.11.

## Results

### Patient characteristics

A total of 377 patients with different liver etiologies were finally included in the analysis, exceeding the required minimum sample size of 101 patients, ensuring robust statistical power for equivalence testing. Patient characteristics are described in Table 1.

**Table 1:** Patient demographics and histological characteristics

Characteristics		Sample (*n* = 377)
Sex, female		232 (61.5%)
Age, years (range)		55 (17‒80)
Body mass index (kg/m^2^)		27.9 ± 5.0
Liver disease etiology	MASLD	205 (54.4%)
	Autoimmune hepatitis	121 (32.1%)
	Alcohol consumption	25 (6.6%)
	Viral hepatitis	18 (4.8%)
	Hemochromatosis	4 (1.1%)
	Other	4 (1.1%)
Histological fibrosis stage	F0	132 (35.0%)
	F1	83 (22.0%)
	F2	79 (21.0%)
	F3	48 (12.7%)
	F4	35 (9.3%)
Histological steatosis grade	S0	174 (46.2%)
	S1	70 (18.6%)
	S2	59 (15.6%)
	S3	74 (19.6%)
Histological iron grade	Fe0	303 (80.4%)
	Fe1	22 (5.8%)
	Fe2	17 (4.5%)
	Fe3	20 (5.3%)
	Fe4	15 (4.0%)

*MASLD* Metabolic dysfunction-associated steatotic liver disease

### Segmentation masks

**Fig. 2:**
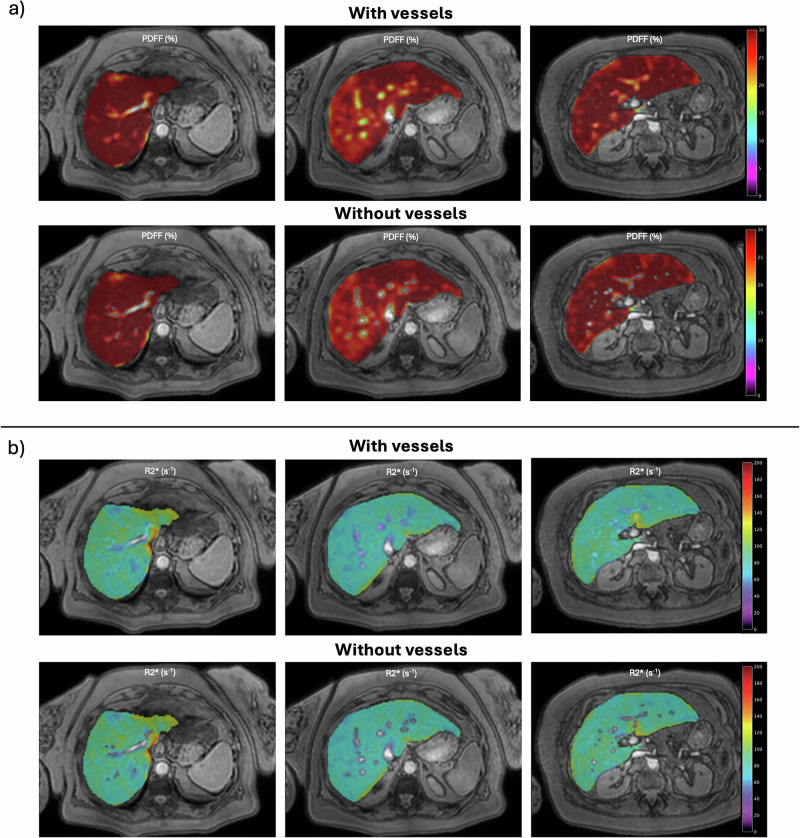
PDFF (**a**) and R2* (**b**) parametric maps from a patient with high steatosis (MASLD S3 histological steatosis grade). For each, the results of the automatic segmentation on three representative slices, including (upper row) and excluding (bottom row) the hepatic vessels, can be observed. Median PDFF: 29.46% with vessels, 29.72% without vessels. MASLD, Metabolic dysfunction-associated steatotic liver disease; PDFF, Proton density fat fraction

**Fig. 3:**
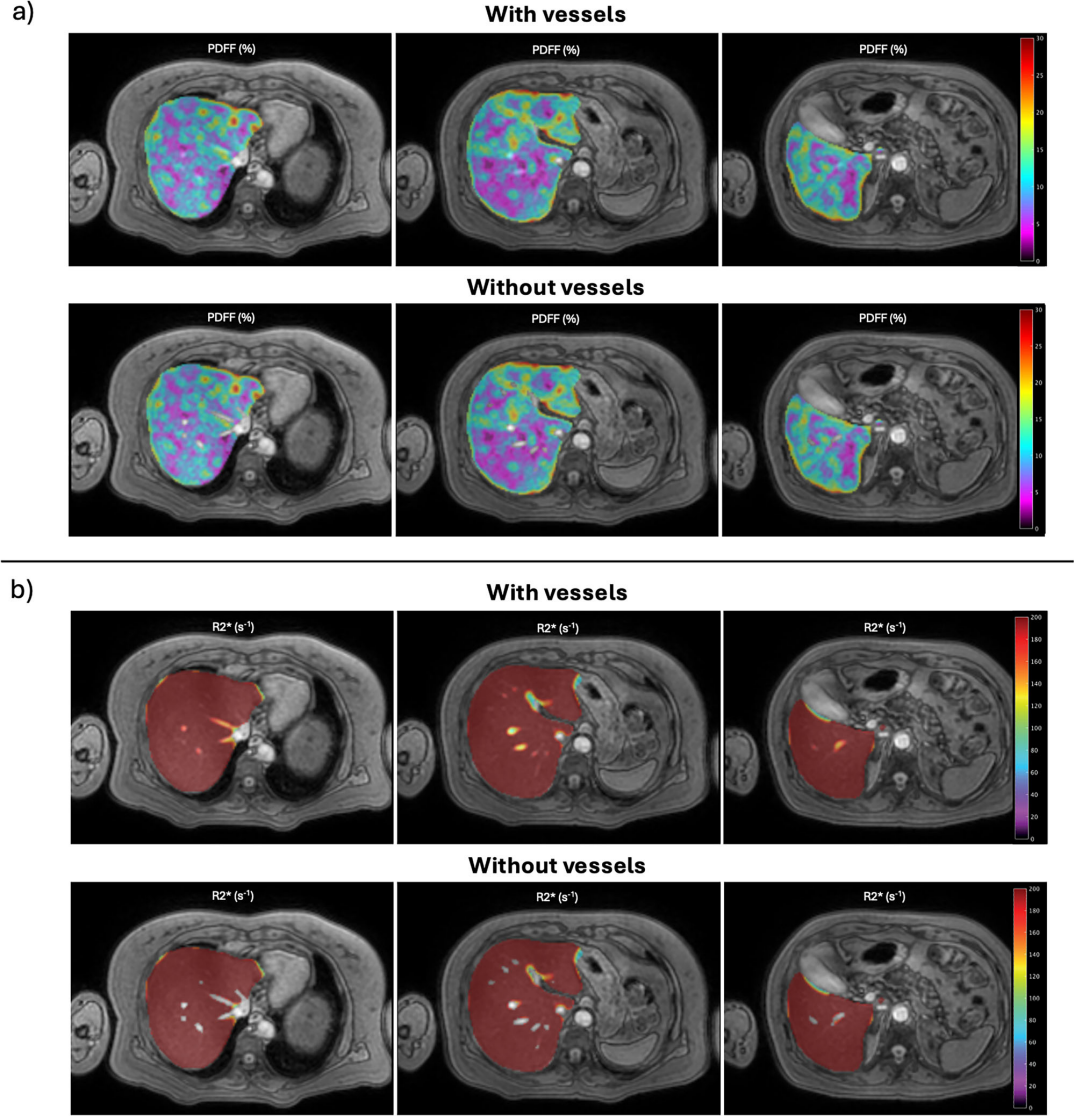
PDFF (**a**) and R2* (**b**) parametric maps from a patient with high iron overload (hemochromatosis Fe4 histological iron grade). For each, the results of the automatic segmentation on three representative slices, including (upper row) and excluding (bottom row) the hepatic vessels, can be observed. Median R2*: 277.42 s^-1^ with vessels, 279.03 s^-1^ without vessels. PDFF, Proton density fat fraction

All cases were automatically segmented with both segmentation methods. Figure 2 shows the corresponding PDFF parametric map in a patient with severe steatosis. Figure 3 shows the corresponding R2* parametric map in a patient with high iron overload. Overall, the population mean liver volume was 1.39 ± 0.37 L, considering the entire liver mask and 1.36 ± 0.36 after excluding the hepatic vessels. The mean vessel volume was 25.52 ± 12.76 mL (1.81 ± 0.64% of the entire liver mask). Across the entire cohort, PDFF and R2* showed minimal absolute variations between both segmentation strategies, with no statistically significant differences (Table 2) and a very strong correlation between segmentation methods (ρ ≥ 0.99).

**Table 2:** PDFF and R2* values (median and interquartile range) extracted from the whole-liver segmentation, including and excluding the hepatic vessels

Metric	With vessels	Without vessels	Absolute difference	*p*-value	ρ
PDFF (%)	3.49 (0.37–10.77)	3.56 (0.38–10.88)	0.06 [0–0.27]	0.90	0.99
R2* (s^-1^)	49.07 (41.59–59.14)	49.27 (41.73–59.41)	0.25 [0–2.93]	0.76	0.99

The absolute difference is represented as the mean [minimum–maximum]

*PDFF* Proton density fat fraction

**Fig. 4:**
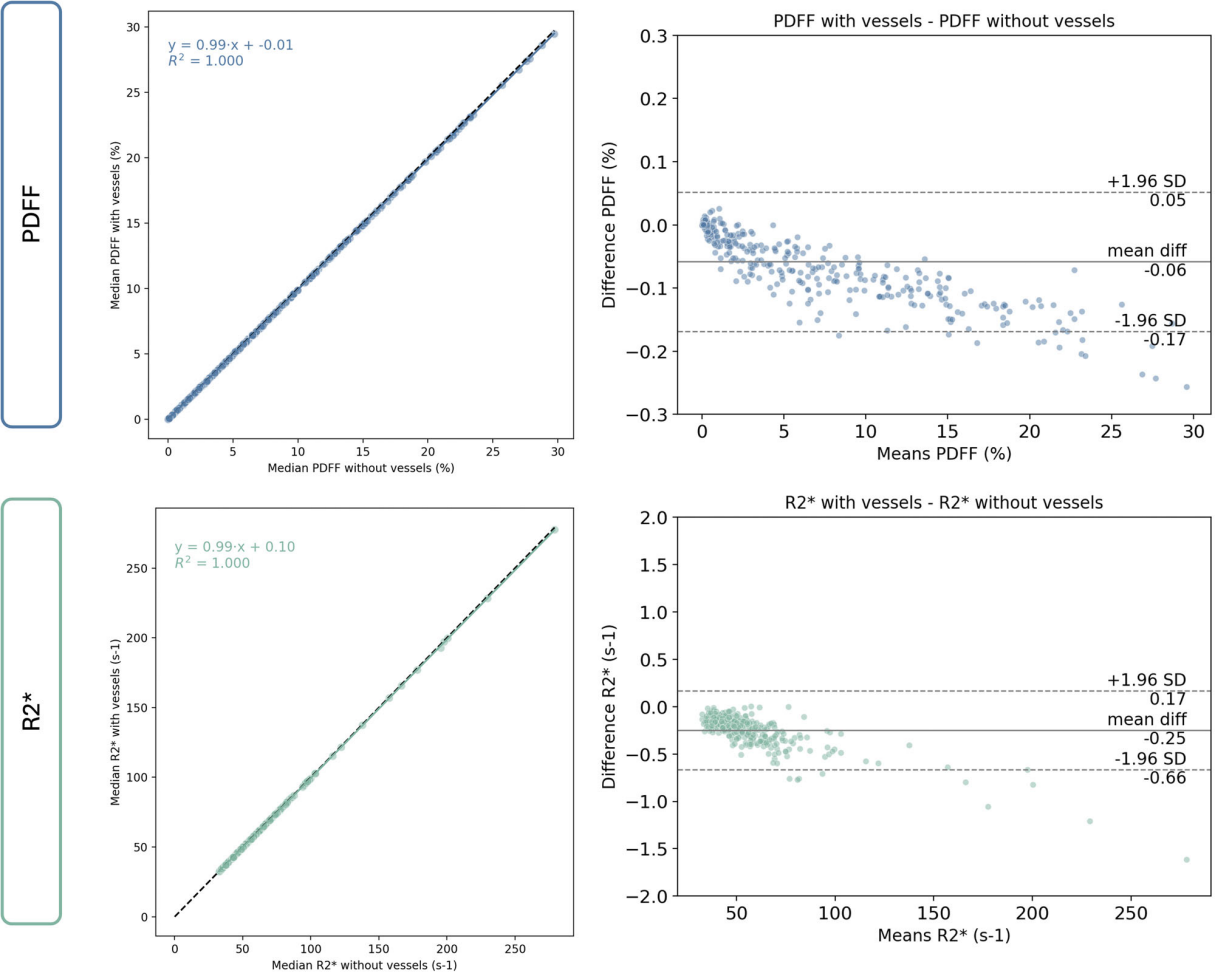
Linear regression (left graphs) and Bland–Altman (right graphs) analysis comparing PDFF (upper row) and R2* (bottom row) values, including the hepatic vessels *versus* excluding hepatic vessels. For regression plots, the solid line represents the fitted regression line, and the dashed line represents the unity line. For Bland–Altman plots, the solid line represents the mean differences (bias), and the dashed lines represent 95% confidence intervals. PDFF, Proton density fat fraction

**Fig. 5:**
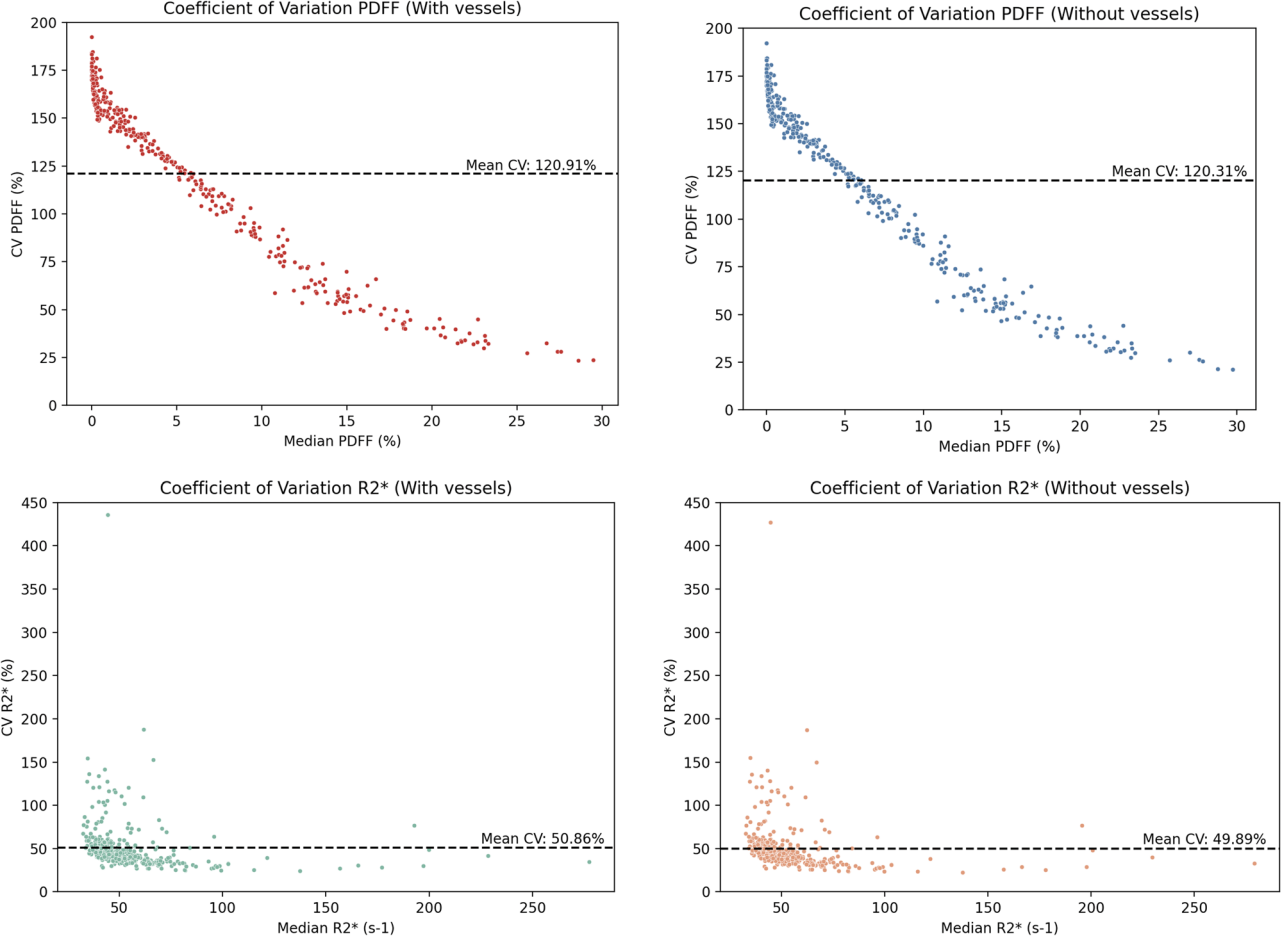
CoV at a case level of PDFF (upper row) and R2* (bottom row) values, including (left graphs) and excluding (right graphs) the hepatic vessels. The dashed line represents the mean CoV value. The outlier present in the R2* CoV analysis corresponds to a case with air in the colon, a small portion was incorrectly included in the automatic segmentation, significantly increasing the R2* standard deviation for both masks. CoV, Coefficient of variation; PDFF, Proton density fat fraction

Linear regression analysis showed a slope of 0.99 for both PDFF and R2*. Bland–Altman analysis showed a minor negative bias when including the hepatic vessels, with a bias of -0.06% for PDFF and -0.25 s^-1^ for R2* (Fig. 4). The CoV analysis at a case level (Fig. 5) showed a lower mean CoV in both PDFF (120.31% *versus* 120.91%) and R2* (49.89% *versus* 50.86%) metrics derived when hepatic vessels were excluded from the segmentation mask.

### Steatosis and iron grades

**Fig. 6:**
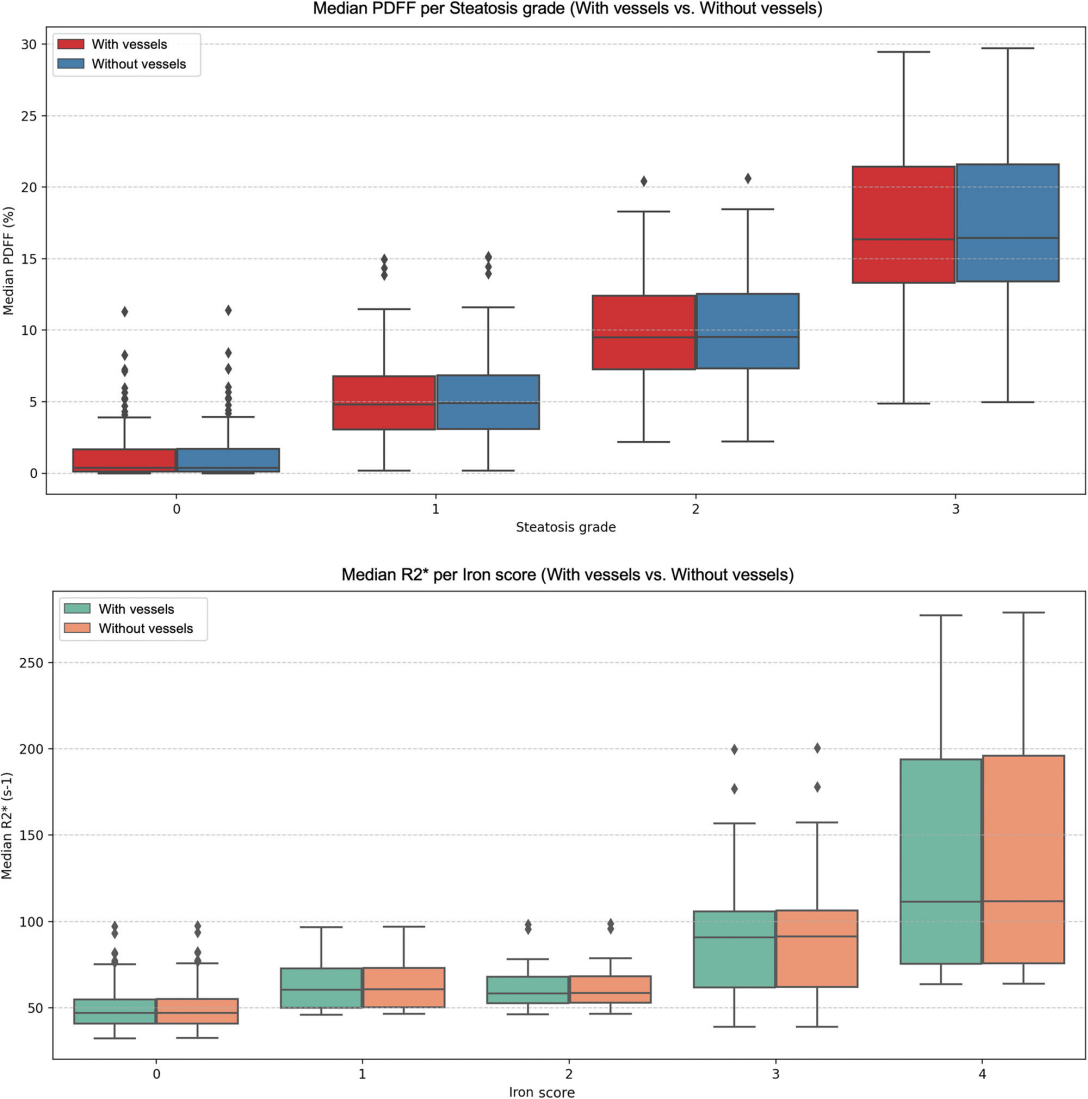
Median PDFF (upper row) and R2* (bottom row) distributions across different histological steatosis and iron overload grades using the liver segmentation, including and excluding the hepatic vessels. PDFF, Proton density fat fraction

After excluding vessels, median PDFF values increased marginally across all histological steatosis grades (S0-S3) (Table 3). Differences between segmentation strategies were not statistically significant, with a very strong correlation between both methods (ρ ≥ 0.99) across each histological grade. Similarly, median R2* values showed minimal changes across histological iron scores, with no statistical differences between the two segmentation masks (Table 3). There was a very strong correlation between both methods (ρ ≥ 0.99) stratified by histological iron overload grades. Figure 6 represents PDFF and R2* values stratified by histological grades with both segmentation masks, showing similar distributions across each grade of steatosis and iron overload.

**Table 3:** PDFF and R2* values (median and interquartile range) extracted from the whole-liver segmentation, including and excluding the hepatic vessels, for each steatosis and iron grade

Histological steatosis grade	PDFF with vessels (%)	PDFF without vessels (%)	*p*-value	ρ
S0 (*n* = 174)	0.36 (0.11–1.67)	0.38 (0.10–1.70)	0.95	0.99
S1 (*n* = 70)	4.82 (3.05–6.78)	4.90 (3.10–6.85)	0.81	0.99
S2 (*n* = 59)	9.48 (7.27–12.41)	9.53 (7.33–12.54)	0.91	0.99
S3 (*n* = 74)	16.15 (13.20–21.41)	16.26 (13.29–21.55)	0.89	0.99
**Histological iron grade**	R2* with vessels (s^**-1**^**)**	R2* without vessels (s^**-1**^**)**	***p*-value**	**ρ**
Fe0 (*n* = 303)	47.02 (40.83–54.96)	47.12 (41.03–55.26)	0.76	0.99
Fe1 (*n* = 22)	60.43 (50.15–72.78)	60.84 (50.43–73.04)	0.95	0.99
Fe2 (*n* = 17)	58.38 (52.81–68.11)	58.54 (52.98–68.42)	0.73	1.00
Fe3 (*n* = 20)	90.83 (61.81–105.90)	91.33 (62.07–106.37)	0.98	0.99
Fe4 (*n* = 15)	111.40 (75.52–193.74)	111.77 (75.86–196.00)	0.98	0.99

*PDFF* Proton density fat fraction

**Fig. 7:**
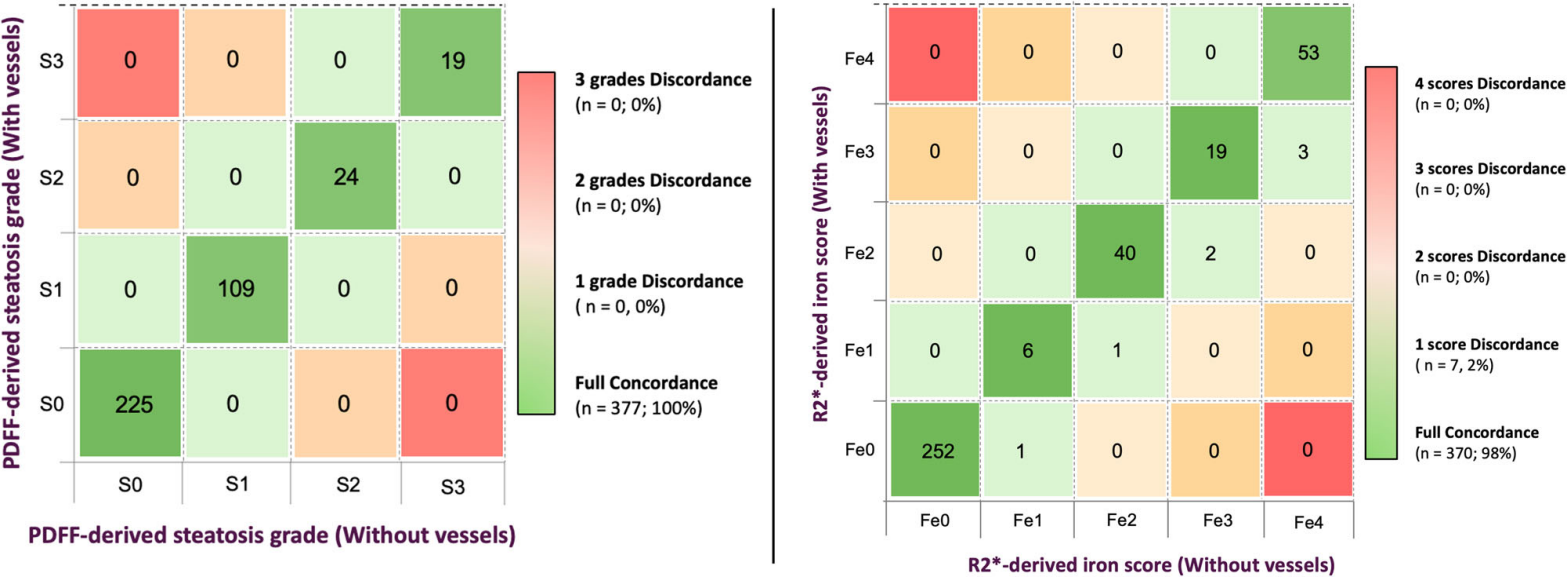
Confusion matrix comparing the PDFF-derived steatosis grades (left) and the R2*-derived iron scores (right) when using the whole-liver segmentation, including and excluding the hepatic vessels. PDFF, Proton density fat fraction

Considering PDFF grading cut-offs, no patients were reclassified into different PDFF grades (PDFF-S0 – PDFF-S3) using the segmentation approach that excludes the vessels. On the other hand, considering R2* grading cut-offs, only seven patients were reclassified, increasing by one grade the iron overload severity after vessel exclusion (Fig. 7). The etiologies of the discordant cases were MASLD (*n* = 5), alcohol related liver disease (*n* = 1), and autoimmune hepatitis (*n* = 1). Supplementary Table S1 shows the median R2* values of these discordant cases before and after vessel exclusion. The difference in liver iron content was very small (≤ 0.55 s⁻¹), and all cases presented R2* values close to the established MRI-derived grade cutoffs. No cases with severe iron overload (Fe4) were misclassified.

## Discussion

This study provides comprehensive evidence that the inclusion of hepatic vessels in the segmentation mask of liver parenchyma has a low impact on PDFF and R2* quantification. Across a large and diverse cohort of patients with chronic liver disease, we observed no statistically significant differences in PDFF or R2* values when comparing segmentation strategies with *versus* without vessels. There was a strong correlation between segmentation methods (ρ = 0.99) and a minimal non-significant absolute difference (≤ 0.27% for PDFF and ≤ 2.93 s⁻¹ for R2*). In addition, the linear regression analysis showed a slope of 0.99 for both imaging biomarkers.

These results have several important clinical implications. First, they provide further validation to current automated whole-liver quantification tools that do not explicitly exclude vessels [11, 15]. In routine clinical practice, where processing speed is crucial and computational algorithms are not uniformly available, our findings support the use of whole-liver masks without complex vessel-exclusion filters as a reliable approach. Second, we demonstrate that vessel inclusion has minimal clinical impact on PDFF and R2* quantification. For PDFF, the absolute differences between segmentation strategies were consistently below the repeatability coefficient of 1.2–1.6% reported by the NIMBLE consortium, which defines the threshold for distinguishing measurement error from true biological change in patients with liver disease [21]. Importantly, no cases showed a change in PDFF-derived steatosis grade regardless of the segmentation method. For liver iron content, the absolute differences between segmentation strategies were below the mean reproducibility bias reported for liver R2* quantification [4]. No cases with severe iron overload were misclassified, and only 7 patients (1.9% of the study population) were reclassified across R2*-derived iron grade. Notably, all reclassified cases had R2* values very close to the thresholds used for categorization into iron overload grades. Unlike PDFF grading, where thresholds span wider ranges, R2* grading is constrained by narrow cut-offs between categories. This explains our findings and highlights the need for future research to establish clinically driven cut-points for liver iron quantification, ideally based on patient outcomes rather than conventional histology. Finally, the hepatic vessel-to-liver volume ratio in chronic liver disease was around 2%, consistent with previous studies [22]. Because this vessel volumetry is small—and even decreases in advanced liver disease [22]—its contribution to whole-liver MRI biomarker quantification is expected to be negligible.

Nonetheless, subtle trends were observed and must be critically considered. Excluding vessels consistently produced slightly higher PDFF and R2* values across all histological grades of steatosis and iron overload. Although these differences were small and not statistically significant, they were directionally consistent, suggesting a systematic effect. In addition, the exclusion of hepatic vessels yielded a lower mean CoV for both PDFF (-0.06%) and R2* (-0.97%). Again, although the magnitude of the change was small, vessel inclusion systematically introduces greater variability relative to the mean (Fig. 3). Bland–Altman analysis further supported this observation, showing a small negative bias when hepatic vessels were included (-0.06% for PDFF and -0.25 s^-1^ for R2*). These differences may be due to the intrinsic properties of blood vessels, since they lack intracellular fat and may have altered magnetic susceptibility due to blood oxygen transportation, leading to distinct signal decay and R2* behavior compared to liver parenchyma [14].

Despite lower mean values and increased CoV, the clinical impact of hepatic vessels inclusion remains negligible, as nearly all cases were classified in the same severity grades determined by MRI. Thus, in hepatology care settings, all patients with moderate-to-severe steatosis or iron overload, will be managed identically regardless of the segmentation strategy used. However, in research settings where maximal measurement precision is required, such as clinical trials for drug development with strict inclusion criteria and longitudinal monitoring, minimizing even small sources of bias becomes desirable [5, 23]. In such contexts, even small reductions in measurement noise can enhance sensitivity to detect changes over time, which is particularly relevant in trials targeting the reduction of MRI biomarkers as a surrogate endpoint [24]. Therefore, while vessel exclusion may not alter routine clinical decisions, it could offer incremental value in research applications where analytical sensitivity is critical.

From a technical perspective, implementing vessel exclusion is feasible and increasingly accessible. Segmentation models based on convolutional neural networks can be trained to distinguish parenchyma from vessels using MRI [25]. Alternatively, intensity-based filtering or shape priors can approximate vascular regions [13, 14]. However, additional processing steps introduce new sources of variability, particularly in patients with aberrant vasculature, large cysts, or tumor involvement. Therefore, segmented regions should always be edited if needed and approved by a radiologist.

From a practical perspective, vessel exclusion may require additional computational steps, which modestly increase processing complexity compared to simple whole-liver segmentation. While modern artificial intelligence tools make this feasible with limited additional time, the balance between improved precision and workflow simplicity should be considered when implementing these methods in routine clinical practice.

A major strength of this study is its generalizability. The patient cohort covers a wide range of liver disease etiologies, and disease severity of histological steatosis and iron grades. The use of close histological reference standards provides a robust anchor for interpreting imaging results. Several limitations merit consideration. First, the study was conducted using a single post-processing platform, although its methodology has been validated across multiple scanners and regions [11]. Second, while we evaluated PDFF and R2*, other MRI biomarkers, such as T1 mapping, extracellular volume, fibrosis, or elastography, may be differentially affected by vascular inclusion and warrant individual analysis. Future studies will be conducted to extend the analysis to a broader set of imaging biomarkers to assess whether the patterns observed for PDFF and R2* generalize to other quantitative MRI parameters, with special consideration to elastography. Third, the absence of pediatric patients in our cohort limits the extrapolation of our findings to children. However, our results are generalizable to adult patients with chronic liver disease, who represent the primary target population for the clinical application of PDFF and R2*. A strength of our study is the inclusion of patients with diverse etiologies and a broad range of disease severity, which enhances the robustness and applicability of the results. Fourth, the slice thickness of 6–7 mm may have introduced partial volume effects, particularly at vessel boundaries, potentially attenuating differences between segmentation strategies.

Finally, the evolving regulatory landscape for imaging biomarkers must be considered. As PDFF and R2* move toward qualification as surrogate endpoints in clinical trials and potentially regulatory approval, standardizing image acquisition and analysis, including decisions around vessel inclusion, will become increasingly important. Consensus guidelines from professional societies such as the European Imaging Biomarkers Alliance—EIBALL and Quantitative Imaging Committee—QUIC may benefit from incorporating evidence from studies like ours to guide best practices.

In conclusion, this study demonstrates that major hepatic vessels have a minimal influence on automated PDFF and R2* quantification using whole-liver segmentation. Additionally, the exclusion of hepatic vessels may enhance clinicians’ trustworthiness and confidence when using these automatic tools in clinical practice.

## Supplementary information

ELECTRONIC SUPPLEMENTARY MATERIAL

## Data Availability

The data that support the findings of this study are available upon reasonable request to the authors (registry number: 2017/0031/PI).

## Abbreviations

CoV: Coefficient of variation
MASLD: Metabolically-dysfunction-associated steatotic liver disease
MECSE: Multiecho chemical shift-encoded
MRI: Magnetic resonance imaging
PDFF: Proton density fat fraction
