# Supplementary material for: Impact of hepatic vessels on whole liver proton density fat fraction and R2* quantification
Source: Eur Radiol Exp. 2026 Jan 5;10:1. doi: 10.1186/s41747-025-00663-1 (PMC12770010; doi:10.1186/s41747-025-00663-1)
Supplement: Supplementary file 1 — ELECTRONIC SUPPLEMENTARY MATERIAL [file 41747_2025_663_MOESM1_ESM.pdf]

Impact of hepatic vessels on whole liver proton density fat fraction and R2* quantification

ELECTRONIC SUPPLEMENTARY MATERIAL

Supplementary Material 1

This section includes the details of the methodology followed and the results obtained for the automatic liver segmentation algorithm excluding the hepatic vessels. The strategy followed was to develop an algorithm for hepatic vessels segmentation and exclude this region from the entire liver organ mask.

Annotation

The dataset used for model development and evaluation was the same as that presented in [1]. Four different data sources were used for model development and validation. Data from two sources using Philips 3T scanners was used for training (n=107) and testing (n=46). The remaining data sources, with MR exams from two different scanners (Philips 3T and Siemens 1.5T) were used for external testing (n=29). Hepatic vessels were segmented on each case from the training and tests datasets by two radiologists with over 5 years' experience in abdominal MR imaging.

The annotations process was performed as followed:

- First, the first echo time image from the MECSE-MR exam was selected and adjusted appropriately to effectively distinguish the vessels from the tissue.
- To identify the vessels, the initial focus lied on the inferior vena cava. Then, particular attention was put on the liver hilum, home to the hepatic portal vein, hepatic artery, and their associated vessels.
- Two factors aided the distinction between vessels from tissue. Firstly, the vessels' pixel intensity was higher and markedly differed from that of liver parenchyma. Additionally, the continuity of the vessels along adjacent slices was used.

Training

The Keras-unet-collection library [2] was used for model implementation. A 2D U-Net-based architecture composed by five convolutional blocks on each branch was used. The number of filters used across the convolutional blocks were 16, 32, 64, 128, and 256. Batch normalization was used after each convolutional layer. The number of convolutional layer per downsampling level were 2, while those per upsampling level were 1. Unpooling was perdomed with nearest neighbour interpolation. The training was performed along 75 epochs with a batch size of 32 images. The loss function to optimize on each iteration was based on the Dice coefficient (DC). The ADAM optimizer was used during the training process. The initial learning rate was set to 1e-3. A 5-fold cross validation strategy was followed. The model saved on each fold was the one with the lowest loss score on the validation set once training is over.

Inference and evaluation

An ensemble including the resulting five models of the cross-validation process was built. Given a new input image, inference was performed on each of the five models, and the final probability map was the result of the averaged probability map. To obtain a binary vessel mask, a threshold of 0.5 was applied. Finally, the segmented area was removed from the automatically generated liver masks using the algorithm present in [1].

For model’s evaluation, first, the DC of the liver vessels mask was computed, then the DC on liver segmentation was calculated, therefore, manually delineated vessels masks were excluded from the liver masks from [1] and used as ground truth.

Results

Considering only the vessels mask, a mean DC of 0.74 was obtained on the test set. Regarding liver mask without vessels, a mean DC of 0.97 was obtained on the internal test set (n=49) composed of cases from the same institutions as those used for training (Philips 3T scanner). On the external test sets a mean DC of 0.93 was obtained on the independent Siemens 3T scanner while a mean DC of 0.85 was observed on the Philips 1.5T scanner.

References

1. Jimenez-Pastor A, Alberich-Bayarri A, Lopez-Gonzalez R, Marti-Aguado D, França M, Bachmann RSM, Mazzucco J, Marti-Bonmati L (2021) Precise whole liver automatic segmentation and quantification of PDFF and R2* on MR images. Eur Radiol. 31(10):7876-7887. https://doi.org/10.1007/s00330-021-07838-5.
2. Sha Y., 2021: Keras-unet-collection. GitHub repository, accessed 4 September 2021, https://doi.org/10.5281/zenodo.5449801

Table S1. Median R2* values before and after vessels exclusion with associated R2*-derived iron scores of the seven reclassified iron overload cases.

**R2* with vessels (s^{-1})**	**R2* without vessels (s^{-1})**	**R2* difference without – with vessels (s^{-1})**	**R2*-derived iron score with vessels**	**R2*-derived iron score without vessels**
68.95	69.14	0.19	Fe3	Fe4
68.92	69.47	0.55	Fe3	Fe4
68.72	69.06	0.34	Fe3	Fe4
63.98	64.29	0.31	Fe2	Fe3
63.73	64.10	0.37	Fe2	Fe3
55.92	56.20	0.28	Fe1	Fe2
54.62	55.03	0.41	Fe0	Fe1
